# Development of a stakeholder-informed framework for the implementation of surgical sabermetrics to enhance training and education

**DOI:** 10.1093/bjs/znag009

**Published:** 2026-02-10

**Authors:** Lachlan Dick, Emma Howie, Joel Norton, Connor Boyle, Andrew Merriman, Victoria Ruth Tallentire, Roger D Dias, Douglas S Smink, Richard J E Skipworth, Steven Yule

**Affiliations:** Surgical Sabermetrics Laboratory, Usher Institute, University of Edinburgh, Edinburgh, UK; Medical Education Directorate, Royal Infirmary of Edinburgh, NHS Lothian, Edinburgh, UK; Clinical Surgery, University of Edinburgh, Edinburgh, UK; Surgical Sabermetrics Laboratory, Usher Institute, University of Edinburgh, Edinburgh, UK; Clinical Surgery, University of Edinburgh, Edinburgh, UK; Surgical Sabermetrics Laboratory, Usher Institute, University of Edinburgh, Edinburgh, UK; Clinical Surgery, University of Edinburgh, Edinburgh, UK; Surgical Sabermetrics Laboratory, Usher Institute, University of Edinburgh, Edinburgh, UK; Clinical Surgery, University of Edinburgh, Edinburgh, UK; Medical Education Directorate, Royal Infirmary of Edinburgh, NHS Lothian, Edinburgh, UK; Medical Education Directorate, Royal Infirmary of Edinburgh, NHS Lothian, Edinburgh, UK; Medical AI & Cognitive Engineering (MAICE) Lab/STRATUS Center for Medical Simulation, Department of Emergency Medicine, Mass General Brigham, Harvard Medical School, Boston, Massachusetts, USA; Department of Surgery, Brigham and Women’s Hospital/Harvard Medical School, Boston, Massachusetts, USA; Surgical Sabermetrics Laboratory, Usher Institute, University of Edinburgh, Edinburgh, UK; Clinical Surgery, University of Edinburgh, Edinburgh, UK; Surgical Sabermetrics Laboratory, Usher Institute, University of Edinburgh, Edinburgh, UK; Clinical Surgery, University of Edinburgh, Edinburgh, UK

## Abstract

**Background:**

Surgical training relies heavily on subjective performance evaluation, which is resource-intensive and prone to assessor bias. Advances in digital surgery offer opportunities for objective assessment. While validity evidence for data-driven assessments increases, strategies for implementation in surgical training remain scarce. The aim of this study was to leverage stakeholder insights to develop an implementation framework for integrating data-driven surgical sabermetrics into training curricula.

**Methods:**

Structured workshops were conducted at two international surgical conferences (the Association of Surgeons of Great Britain and Ireland Congress, Edinburgh, May 2025 and the International Conference on Surgical Education and Training, Edinburgh, June 2025). Delegates participated in facilitated discussions, interactive polling, and group concept-mapping exercises to explore opportunities, delivery modalities, access rights, and contextualization for surgical performance metrics. Stakeholder perceptions were used to iteratively develop an implementation framework, balancing applicability to current training pathways and capturing the nuances of data-driven insights.

**Results:**

A total of 54 surgical trainees and trainers from 13 countries contributed. Opportunities centred on enhancing objective feedback, assessing non-technical skills, and tracking trainee progression. Video-based delivery and real-time feedback were prioritized for technical skills, dashboards were prioritized for non-technical and cognitive skills, and structured reports were prioritized for performance-based metrics. Supervising surgeons and training leads were identified as essential users of trainee data, with integration of multimodal data (for example surgeon physiology, case complexity) deemed essential for contextualization.

**Conclusion:**

This study presents an implementation framework for surgical sabermetrics in training. The framework provides practical guidance on delivery, access, and integration of performance metrics, supporting data-driven feedback to optimize trainee development, advance surgical education, and improve patient outcomes.

## Teaser

Data-driven surgical sabermetrics presents opportunities to advance training through enhanced insights, reliability, and objectivity. Identifying optimal delivery modalities, access rights, and data for contextualization is critical to realize the potential. Guided by stakeholder insights, this study proposes a framework for implementing surgical sabermetrics to enhance training and education.

## Introduction

The operating theatre is one of the highest-stakes environments in healthcare. Despite advances in perioperative care, surgical complications continue to impose significant burdens on patients and healthcare systems^1^. Although surgical outcomes are multifactorial, the influence of technical skills (for example tissue handling) and non-technical skills (for example situation awareness) is well established^2,3^. Greater focus on these domains, delivered through enhanced education and training, could better equip surgeons and teams to deliver high-quality care and improve outcomes. However, current appraisal methods rely on subjective opinion, which is resource-intensive, often lacks precision, and is prone to observer bias^4^. There is an urgent need to adopt reliable and objective methods for analysis and feedback of clinicians’ operative performance^5^.

The increasing digitization of surgery has great potential to transform current practice^6^. From video feed in minimally invasive surgery^7^, cognitive load measurement of surgical teams via wearable sensors^8^, and artificial intelligence (AI)-powered ‘black box’ technology^9^, a wealth of data generated in the operating theatre can be leveraged. Video-based analytics have been deployed in real-world settings in both North America and Europe^10,11^ and it has been shown that derived performance metrics can accurately discriminate skill level^7^. The feasibility of using sensors to assess clinical performance has been demonstrated^12^ and the relationship of sensor outputs (for example surgeon heart-rate variability (HRV)) with patient outcomes has been established^13^. The emerging field of surgical sabermetrics combines audiovisual and sensor data with advanced analytics to provide enhanced insights and support professional development^14^. Inspired by data-driven performance enhancement in professional sports, surgical sabermetrics mirrors the approaches used to guide player evaluation and team-level tactical decisions. Applying these principles in surgery could address the limitations in current practice (for example observer bias) and lead to more effective utilization of data (for example automated segmentation and evaluation of video).

To date, the focus of sabermetrics has been on technology development and validation, with limited exploration into the strategies needed for implementation into surgical curricula^7^. The authors’ previous Delphi consensus study established the need for data-driven insights to reduce variability in assessments and identified ten specific metrics essential in a training context (for example technical errors, situation awareness)^15^. However, there remains a relative paucity of literature to propose how this consensus should be realized^15^. Optimal delivery modalities, data-access rights, and supporting material for contextualization remain poorly defined. This gap has emerged as a key priority for surgeons, researchers, and education leaders^16,17^. A systematic strategy would provide consistency in approach, ensuring that emerging technologies can be embedded within training in a coherent and sustainable manner. To develop an implementation framework, the present study employed stakeholder workshops to inform a blueprint for the delivery, access, and contextualization of data-derived sabermetrics within surgical training.

## Methods

**Fig. 1:**
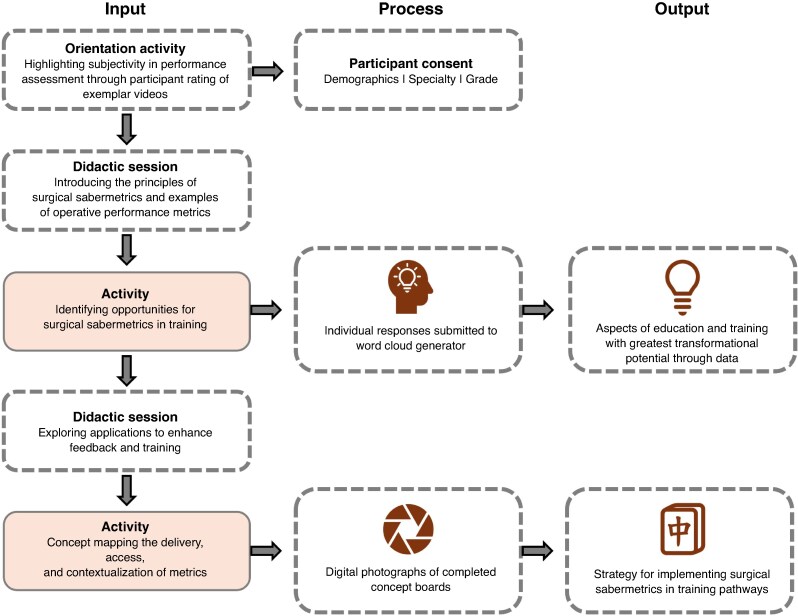
Flow diagram of the workshop structure and data collection

This study leveraged interactive workshops to capture the broad insights of stakeholders on the implementation of surgical sabermetrics. Three workshops were held at two international surgical conferences: the Association of Surgeons of Great Britain and Ireland Congress, Edinburgh, May 2025 (one workshop) and the International Conference on Surgical Education and Training, Edinburgh, June 2025 (two workshops). Each 90-min workshop facilitated group-based discussion and concept mapping to explore opportunities and requirements for the use of sabermetrics in surgical training (*Fig. 1*). This work was overseen by a multidisciplinary steering group, comprising trainees, surgeons, educators, and data-science researchers, who guided the design and delivery of the workshops and provided oversight of data collection and analysis. As the primary aim was a stakeholder-driven exercise to generate insights for an emerging field, an underlying implementation theory was not applied.

Approval for this study was granted by the Edinburgh Medical School Research Ethics Committee (reference: 24-EMREC-020_A2). Participation was voluntary. No inclusion/exclusion criteria were used, enabling all conference delegates to participate. Data were collected using three digital tools: the Research Electronic Data Capture (REDCap) platform (for consent and demographic data)^18^, Wooclap (Brussels, Belgium (wooclap.com); for anonymous polling), and digital photographs (to capture participant concept boards).

### Data capture

After providing informed consent, anonymous demographic data were collected, including sex, specialty, grade, and country of practice. A short orientation lecture introduced the fundamentals of surgical sabermetrics. Participants were shown the role of video analysis and sensor use to measure surgeon performance, with real-world examples used to provide further context. The orientation was delivered by the same three members of the steering group (L.D., E.H., and J.N.) at each workshop to maintain consistency.

Participants first contributed insights into opportunities for integrating sabermetrics into surgical training using an interactive word cloud generator (Wooclap). Responses were collected using a waterfall technique to reduce herd bias, displayed in real time as an aggregated word cloud, and discussed using a ‘one-two-four-all’ technique^19^. After this, participants could provide further suggestions based on group discussions. All free-text responses were extracted for post-hoc qualitative analysis.

Participants then completed group concept-board exercises to determine the optimal delivery, presentation, access rights, and data integration for ten predefined metrics (*Table 1*), identified from the authors’ recent pan-specialty, international Delphi consensus study^20^. Delivery and presentation encompassed the way in which trainees would receive feedback (for example via an interactive dashboard or haptic feedback) and how it would be framed (for example with or without peer benchmarking). Access rights involved determining which individuals would be able to view trainee performance metrics. Data integration involved determining the additional data streams and sources that might be needed to provide a more holistic understanding of the primary performance metric (for example a technical error being supported by information regarding case complexity). Groups were given concept cards for each category and were asked to match performance metrics with examples of delivery and presentation, access rights, and data integration. Examples for each category were informed by prior work on audiovisual analysis^7,21^, sensor use^12,22^, and educational practice^5,23^. Facilitators guided discussion and an example concept board was shown for reference (*Fig. S1*). On completion of the activity, digital photographs were taken of each completed concept board to enable post-hoc analysis of results.

**Table 1:** Concept cards provided to each group of participants

Metrics	Delivery and presentation	Access rights	Data integration
**Technical skills** Technical errors Economy of motion Dissection in the correct tissue plane **Non-technical skills** Situation awareness Decision-making Communication Cognitive load **Performance-based** A safety score A global performance score Duration to react to adverse events	**Delivery** Interactive chart Comprehensive dashboard Audio feedback Annotated image of surgical field Video (for example with overlying heatmap) Structured report (including expert comments) Haptic Numerical (for example score card) Infographic **Presentation** Personal performance only Benchmarked against peers Linked with training portfolio assessment Web-based platform Smartphone-based platform	Trainee only Trainer (for example supervising surgeon) Clinical supervisor Educational supervisor Training director Department clinical lead Hospital management Patient	Operating-theatre audio Video of operating theatre Video of surgical field HRV Radiology images Case complexity score Electrodermal activity Electroencephalography Eye-tracking data fNIRS

HRV, heart-rate variability; fNIRS, functional near-infrared spectroscopy.

### Analysis and development of the implementation framework

To identify opportunities for surgical sabermetrics in training, individual responses were analysed and grouped into themes by a single member of the steering group (L.D.) using an inductive thematic approach. Themes were then mapped to represent trainee, trainer, or mutual opportunity.

For each metric included in a concept board, the corresponding suggestions for delivery and presentation, access rights, and data integration were extracted independently from digital photographs by two coders (L.D. and E.H.) and entered into a structured spreadsheet. The suggested time to use a metric (for example before surgery, after surgery) for training purposes was also extracted. Inter-rater agreement was assessed for each category using Cohen’s κ, with disagreements settled via discussion between the two coders^24^. Frequency mapping was performed to quantify the prominence of each response across individual metrics (for example economy of motion) and broader domains (for example technical and non-technical skills). Framework development proceeded iteratively, drawing on: domain-level frequency trends from concept mapping; contemporaneous facilitator notes from each workshop; and discussion among the steering group. Initial framework components were identified through thematic grouping of recurrent concepts. These were refined over successive rounds of review, during which overlapping elements were merged and rarely cited elements were discarded.

The resulting framework was designed to maximize clarity and applicability for educators while retaining sufficient detail to capture the nuances of data-driven sabermetric insights in surgical training.

## Results

### Participants

A total of 54 participants were recruited across the three workshops. The majority were male (35 participants (65%)) and practiced in the UK (38 participants (70%)). Overall, 13 countries were represented. Of the participants, 27 (50%) were consultant surgeons, 12 (22%) were surgical trainees, 6 (11%) worked in academia (for example as faculty educators), 5 (9%) were senior surgical fellows, and 4 (7%) responded with ‘other’, without providing further detail. The majority of participants were drawn from general surgery (31 participants (57%)). Full demographics are presented in *Table S1*.

### Opportunities for surgical sabermetrics in training

**Fig. 2:**
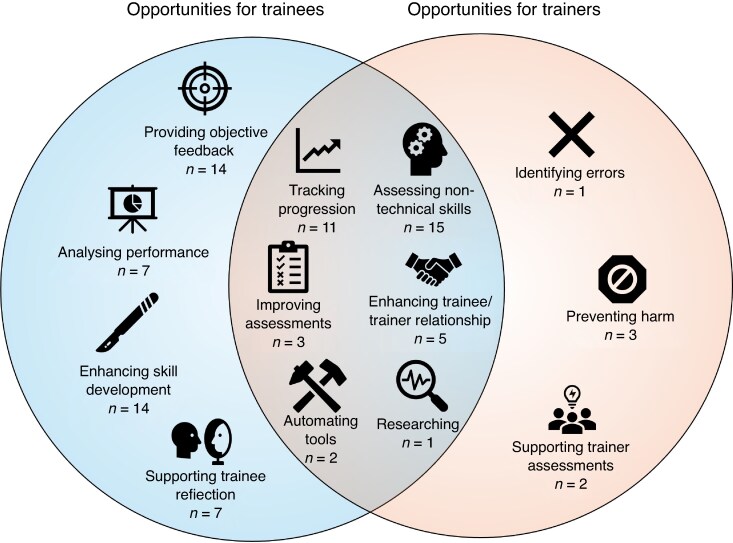
Stakeholder-driven opportunities for surgical sabermetrics in training, demonstrating overlap between priorities for trainers and trainees, with the number of individual responses for each theme given in parentheses

Analysis of the 85 individual responses identified a total of 13 themes (*Fig. 2*). The greatest opportunity identified was in assessing non-technical and cognitive management skills (15 responses). Providing objective feedback (14 responses), enhancing skill development (14 responses), and tracking trainee progression (11 responses) were also prominent themes.

### Implementation framework developed

Alongside these emergent themes, participants engaged with each of the ten performance metrics previously established through the authors’ international Delphi study. Inter-rater reliability for the two coders was strong for delivery and presentation (0.9), access rights (0.85), and data integration (0.82) and moderate for phase of delivery (0.73). Technical errors and a global performance score emerged as the most frequently discussed metrics (both included in 9 concept boards), with situation awareness the most frequently identified non-technical skill metric (included in 8 concept boards). Video-based delivery (suggested 11 times in concept boards), supervising trainer access (suggested 11 times in concept boards), and the integration of HRV data (suggested 8 times in concept boards) were among the most commonly suggested components.

Most concept boards indicated that technical skill metrics should be applied to support real-time feedback (18 suggestions (78%)). Video-based modalities were frequently highlighted for delivery, with access typically required for the supervising surgeon and other clinical supervisors. To ensure meaningful contextualization, both audiovisual data (for example audio from the operating theatre) and trainee physiological sensor data (for example HRV) were deemed necessary.

Metrics relating to non-technical skills and cognitive load management were divided between preoperative preparation (10 suggestions (42%)) and real-time intraoperative feedback (11 suggestions (46%)). While decision-making mirrored the real-time delivery approach for technical skills, other non-technical domains were more commonly paired with interactive visualization dashboards. Again, supervising surgeons were deemed to require access, with additional patient-level data (for example case complexity scores) recognized as important for contextualization.

Performance-based metrics were proposed predominantly for postoperative feedback (16 suggestions (84%)). Suggestions included the use of structured reports integrated within trainee portfolios, enhanced by expert commentary and benchmarking against peers at a similar stage of training. For these metrics, access was generally extended to senior educators or clinical leads (for example training directors, departmental leads), with both audiovisual and patient-specific data recommended to support interpretation.

**Fig. 3:**
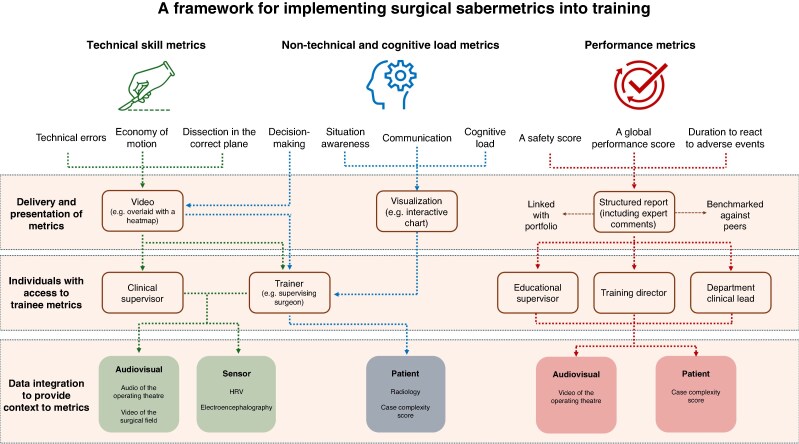
Implementation framework for the integration of surgical sabermetrics into surgical training Each domain (green = technical skill metrics, blue = non-technical and cognitive load metrics, and red = performance metrics) is represented by specific metrics identified from a previous pan-specialty, international Delphi consensus study. For each metric, the proposed flow of delivery, access rights, and contextualization is represented by arrows. For performance metrics, additional arrows highlight the need for portfolio integration and peer benchmarking. The different colours highlight overlapping features between domains. HRV, heart-rate variability.

Using stakeholder insights for delivery, access, and contextualization and the current literature in surgical education, data analytics, and technology integration, the steering group developed the proposed implementation framework (*Fig. 3*).

## Discussion

**Fig. 4:**
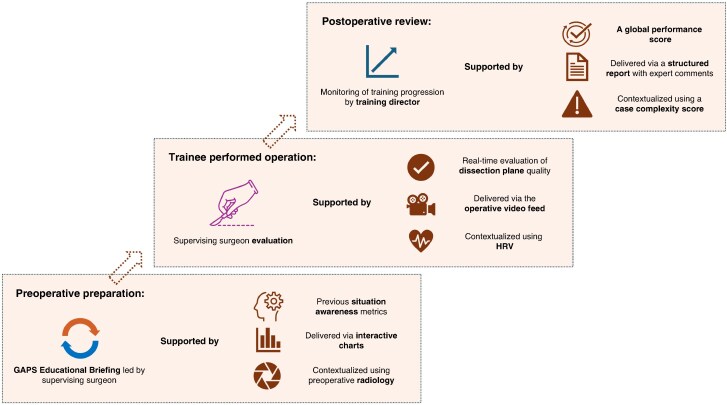
Pipeline of the integration of surgical sabermetrics into perioperative training An existing educational briefing framework (the GAPS framework) is used as an example of existing practice. GAPS, Goals, Autonomy, Preparation, and Strategy; HRV, heart-rate variability.

This study presents a novel, stakeholder-informed framework for the implementation of technology-enhanced surgical sabermetrics into surgical training. The proposed framework identifies unique delivery modalities for each performance domain; real-time visual feedback for technical skills and decision-making, interactive charts for both preoperative preparation and real-time assessment of non-technical skills, and structured reports incorporating peer benchmarking for performance-based metrics. Data-access rights reflect both educational and clinical hierarchies and additional data streams reinforce the need for context-sensitive use of operative performance metrics. Within this framework, success is conceptualized as progressive attainment of competency-based benchmarks aligned to existing curricular expectations, rather than absolute numeric thresholds. Performance is interpreted longitudinally, using sabermetric trends to evaluate improvement across domains. A specific pipeline illustrating how sabermetrics could be integrated into perioperative training, based on the developed framework and utilizing the Goals, Autonomy, Preparation, and Strategy (GAPS) framework for perioperative educational briefing^22^, is shown in *Fig. 4*.

Addressing subjectivity in assessments and providing trainees with cumulative performance appraisal are frequently recognized challenges, which could be addressed by surgical sabermetrics. Previous studies demonstrating that trainees generally feel positive towards receiving data-derived feedback reinforce this potential^10,25^. The prevalence of bias in trainee evaluation with regard to sex and race further emphasizes the need for data-driven insights^4^. By focusing on key attributes in surgery (for example technical and non-technical competencies), surgical sabermetrics could provide trainees with more reliable, trustworthy, and meaningful feedback on performance. To achieve this, wearable devices must be comfortable, physiological differences between the sexes must be recognized, and algorithms must be developed with diverse stakeholders to reduce the risk of embedded bias.

Participants identified assessment of non-technical and cognitive management skills as the area with greatest potential impact. This finding is reflected in the developed framework, where metrics representing these skills are applied both live and before surgery. There is increasing validity evidence to support objective assessment of select non-technical and cognitive skills using physiological measurements. Eye-tracking metrics^26^, HRV^27^, electrodermal activity^28^, and functional near-infrared spectroscopy (fNIRS)^29^ can reliably be used to evaluate cognitive load, with the greatest evidence for HRV. Reflecting the findings of the authors’ consensus study, in a training setting, physiological and cognitive metrics are best suited to guiding longitudinal development, rather than for summative, high-stakes assessment. The aim should be for formative assessment, focusing on user-centred design to ensure that the needs of trainees, trainers, and training directors are met.

The authors’ framework highlights the need for real-time, video-based delivery of technical skill metrics. Augmenting operative video feeds with heatmaps to demonstrate dissection planes has previously been demonstrated in both laparoscopic cholecystectomy and rectal surgery^11,30^. The present study recommends the optimal delivery modality for non-technical and cognitive management skills is through interactive visuals. Dynamic dashboards can incorporate multimodal data streams and are associated with high engagement from surgical trainees^31,32^. Use of these metrics for both preoperative preparation and intraoperative monitoring could prime trainees for potentially challenging cases and support trainers to identify when intervention is required to maintain patient safety (for example if the maximal cognitive load of a trainee is approaching). This proactive approach could also support trainee psychological safety. For performance-based metrics (for example a global performance score), a structured report is required. Crucially, expert-based reflections are essential to provide insights into wider contextual factors (for example interpersonal dynamics) and interpretation of outlying cases (for example due to complexity)^33^.

Governance and access to data captured in the operating theatre remain key concerns^34^. Granting access to supervising surgeons is both pragmatic and necessary to support real-time monitoring of trainee performance. Conversely, individuals with an overview of training progression (for example training directors) need only aggregated measures of global performance. Although patients have expressed interest in accessing operating-theatre data^35^, the proposed framework does not recommend patient access to trainee performance metrics as they require contextual understanding to interpret and this practice may discourage trainees from seeking operative opportunities. It is still necessary to address patient concerns (for example data storage) during the consent process and provide transparency over intended data uses. By focusing on these attributes, patients can develop an understanding of how sabermetrics supports surgeon development and the delivery of safe, high-quality care. Similar consideration is needed for trainee consent. While many engage with wearable technologies in everyday life (for example smartwatches), specific attention is needed around how data are integrated within training pathways.

It is important to recognize that, in some cases (for example intraoperative decision-making), it may not be possible to derive meaningful insights from one data source alone^36^. Providing data on HRV when there are technical errors could lead to the identification of trends and help in the development of mitigating strategies for future cases (for example planned pauses in procedures before phases of high cognitive load). Case complexity (for example disease severity, classification of physical status) could support interpretation of situation awareness metrics and global performance scores and allow underperformance to be evaluated in the context of the overall case. For example, the threshold for expected global performance could be adjusted based on case complexity.

While the present study proposes a framework, implementation requires consideration of barriers and facilitators. Integration with existing curricular pathways is needed to ensure data-derived metrics become integral, rather than isolated adjuncts. The authors’ example pipeline suggests one approach, incorporating operative performance metrics with an existing educational framework. This may not suit all learning styles and so allowing full autonomy on when to deploy these technologies is necessary^10^. Faculty development is essential to support trainers in managing trainee underperformance through reflection, targeted coaching, and goal setting. However, generalizability across different curricula is still needed. Similar to clinical tools introduced on a large scale (for example the Surgical Safety Checklist), the proposed implementation framework could be modified to suit different training settings and environments. This may be driven by legislation requirements (for example for data storage) or lack of digital infrastructure. In such contexts, simple changes in perioperative training practices (for example by using operative video as a feedback tool) could help prime surgeons and trainees for future adoption of data-driven insights. Finally, demonstrating improved training and patient outcomes are future research priorities. Achieving these research priorities will strengthen support from training regulators, surgical colleges, and certification bodies and accelerate adoption of novel performance indicators.

Although the workshops included international representation, most participants were UK-based, male, and drawn from general surgery. Females may conceptualize or value data-related attributes differently, meaning some perspectives may be under-represented. Future work should deliberately broaden stakeholder representation to enhance the generalizability and equity of the proposed framework. Norms of practice in general surgery may have influenced the framework, restricting generalizability. However, conducting the workshops in conference settings enabled engagement with a broad range of stakeholders who were not already known to the study team and conducting the workshops at two independent conferences helped to minimize the influence of a single cohort or a single institution. Due to the nature of participant recruitment, it was not feasible to include patient and public involvement, meaning that patient views may be under-represented (for example on patient access to trainee metrics), highlighting opportunities for future research. Finally, although multidisciplinary, stakeholders were predominately clinical and may have lacked technical knowledge of multimodal data science, resulting in conceptual, rather than practical, discussions that may have over-represented the potential for data-driven surgery. The proposed model requires testing and iterative development, based on the real-world experience of the international surgical training community, to facilitate wider adoption.

Modern surgical training demands reliable, objective assessment methods to support trainee development and enhance patient safety. Leveraging international collaboration, the authors developed a novel, practical implementation framework for integrating operative performance metrics into surgical training. The authors’ future work will explore the real-world application of surgical sabermetrics, guided by this framework, focusing on broader representation of participants and implementation across a range of specialties and training systems. By embedding sabermetric innovations within structured training pathways during real-world applications, surgery can evolve towards more objective, efficient, and impactful feedback, optimizing future surgeons’ progression and advancing the quality and safety of care.

## Supplementary Material

znag009_Supplementary_Data

## Data Availability

Data for this study are not publicly available but may be provided upon reasonable request.
