# Supplementary material for: Development of a stakeholder-informed framework for the implementation of surgical sabermetrics to enhance training and education
Source: Br J Surg. 2026 Feb 10;113(3):znag009. doi: 10.1093/bjs/znag009 (PMC13016916; doi:10.1093/bjs/znag009)
Supplement: znag009_Supplementary_Data [file znag009_supplementary_data.docx]

**Development of a stakeholder informed framework for the implementation of surgical sabermetrics to enhance training and education**

**Supplementary Material**

Lachlan Dick ^1,2,3^, Emma Howie ^1,3^, Joe Norton ^1,3^, Connor Boyle ^1,3^, Andrew Merriman ^2^, Victoria Ruth Tallentire ^2^, Roger D. Dias ^4^, Douglas S Smink ^5^, Richard JE Skipworth ^1,3^, Steven Yule ^1,3^

1. Surgical Sabermetrics Laboratory, Usher Institute, University of Edinburgh, EH16 4UX,

2. Medical Education Directorate, Royal Infirmary of Edinburgh, NHS Lothian, EH16 4SA

3. Clinical Surgery, University of Edinburgh, EH16 4SA

4. Medical AI & Cognitive Engineering (MAICE) Lab / STRATUS Center for Medical Simulation, Department of Emergency Medicine, Mass General Brigham, Harvard Medical School, Boston, MA

5. Department of Surgery, Brigham and Women’s Hospital/Harvard Medical School, Boston, MA

**Corresponding author.** Lachlan Dick. Surgical Sabermetrics Laboratory, Usher Institute, University of Edinburgh, EH16 4UX. [lachlan.dick@ed.ac.uk](mailto:lachlan.dick@ed.ac.uk) **ORCID ID** 0000-0002-4341-9501; **Twitter** @lachiedick

**Supplementary Materials - Index**

| **Supplementary Figures and Tables** |  |
| --- | --- |
| Table S1 | *pag. 2* |
| Figure S1 | *pag. 4* |
|  |  |

**Supplementary Figures and Tables**

**Table S1**

Full participant demographics.

| **Characteristic** | **Number (%)** |
| --- | --- |
| **Gender:** |  |
| Male | 35 (64.8) |
| Female | 18 (33.3) |
| Prefer not say | 1 (1.9) |
| **Country:** |  |
| United Kingdom | 38 (70.2) |
| Australia | 3 (5.4) |
| Belgium | 2 (3.5) |
| Spain | 1 (1.9) |
| Argentina | 1 (1.9) |
| New Zealand | 1 (1.9) |
| Hong Kong | 1 (1.9) |
| Republic of Ireland | 1 (1.9) |
| Malta | 1 (1.9) |
| Malaysia | 1 (1.9) |
| Sweden | 1 (1.9) |
| USA | 1 (1.9) |
| UAE | 1 (1.9) |
| Prefer not to say | 1 (1.9) |
| **Surgical specialty:** |  |
| General surgery | 31 (56.8) |
| Orthopaedics | 5 (9.3) |
| Urology | 4 (7.4) |
| Non-clinician | 3 (5.6) |
| Otolaryngology | 2 (3.8) |
| Vascular | 2 (3.8) |
| OMFS | 2 (3.8) |
| Medical student | 2 (3.8) |
| Dentistry | 1 (1.9) |
| Paediatric | 1 (1.9) |
| Cardiothoracic | 1 (1.9) |
| **Grade:** |  |
| Consultant/Attending | 27 (50) |
| Senior trainee (e.g. registrar) | 8 (14.8) |
| Academic | 6 (11.1) |
| Junior trainee (e.g. core trainee) | 4 (7.4) |
| Other | 4 (7.4) |
| Specialty doctor | 3 (5.6) |
| Fellow | 2 (3.7) |

**Figure S1**


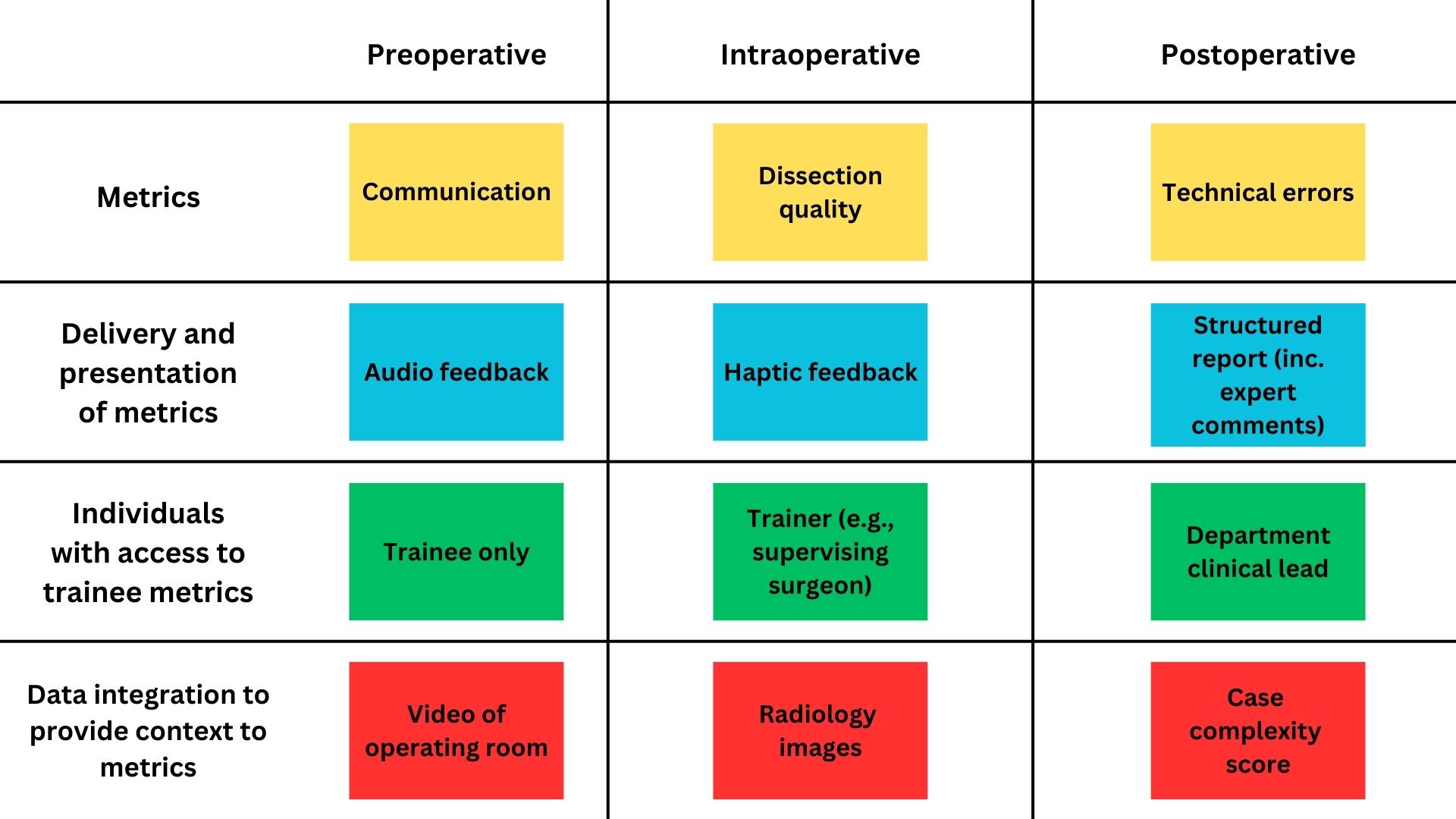


An example concept board shown to participants. The yellow cards reflect individual metrics, with delivery and presentation modalities (blue), access rights (green) and data integration (red) concepts mapped to each metric.
